# Artificial intelligence and sports genomics: advancing precision sports science and athletic performance

**DOI:** 10.3389/fspor.2026.1906206

**Published:** 2026-09-04

**Authors:** Anil Patani, Bhakti Patel, Ramesh Pandit, Snehal Bagatharia, Ashish Patel

**Affiliations:** 1 Gujarat Biotechnology Research Centre (GBRC), Gandhinagar, India; 2 Department of Life Sciences, Hemchandracharya North Gujarat University, Patan, India; 3 Gujarat State Biotechnology Mission (GSBTM), Gandhinagar, India

**Keywords:** artificial intelligence, athletes performance, genetic markers, injury risk, sports genomics

## Abstract

The integration of artificial intelligence (AI) and genomics in sports science has immense potential in the field by enabling personalised, predictive, and data-driven approaches to enhance athletes' performance and mitigate injury risk. Traditional training models based on generalised protocols may overlook individual variations in training adaptation, recovery, and injury susceptibility. Recent breakthroughs in genomics, wearable technologies, multi-omics profiling, and machine learning have produced new possibilities for precision sports medicine. This review explores the molecular and physiological foundations of athletic performance, highlighting the influence of key genetic polymorphisms, such as *ACTN3*, *ACE*, *PPARGC1A*, and collagen-related genes, on endurance, strength, metabolism, and injury vulnerability. The review additionally analyses the use of AI technologies, including machine learning, deep learning, predictive analytics, and systems biology approaches, in analysing complex physiological, biomechanical, and genomic datasets. The applications of AI-driven individualised training, nutrigenomics, biomarker-guided recovery, wearable sensor technologies, and injury prediction models are thoroughly reviewed. The integration of genomics with AI-driven predictive algorithms may support athlete classification, training optimisation, and assessment of fatigue and musculoskeletal injuries through polygenic risk score and multi-omics analysis. Concerns regarding ethics and legality related to genetic privacy, data security, discrimination, and the misuse of genome technology in sports are also highlighted. Although significant obstacles such as limited reproducibility, small sample numbers, population bias, and difficulties in data integration remain serious limitations, future developments in single-cell omics, digital twins, and real-time biosensing technologies may further contribute to precision sports science and personalised athlete management.

## 1 Introduction

**Figure 1:**
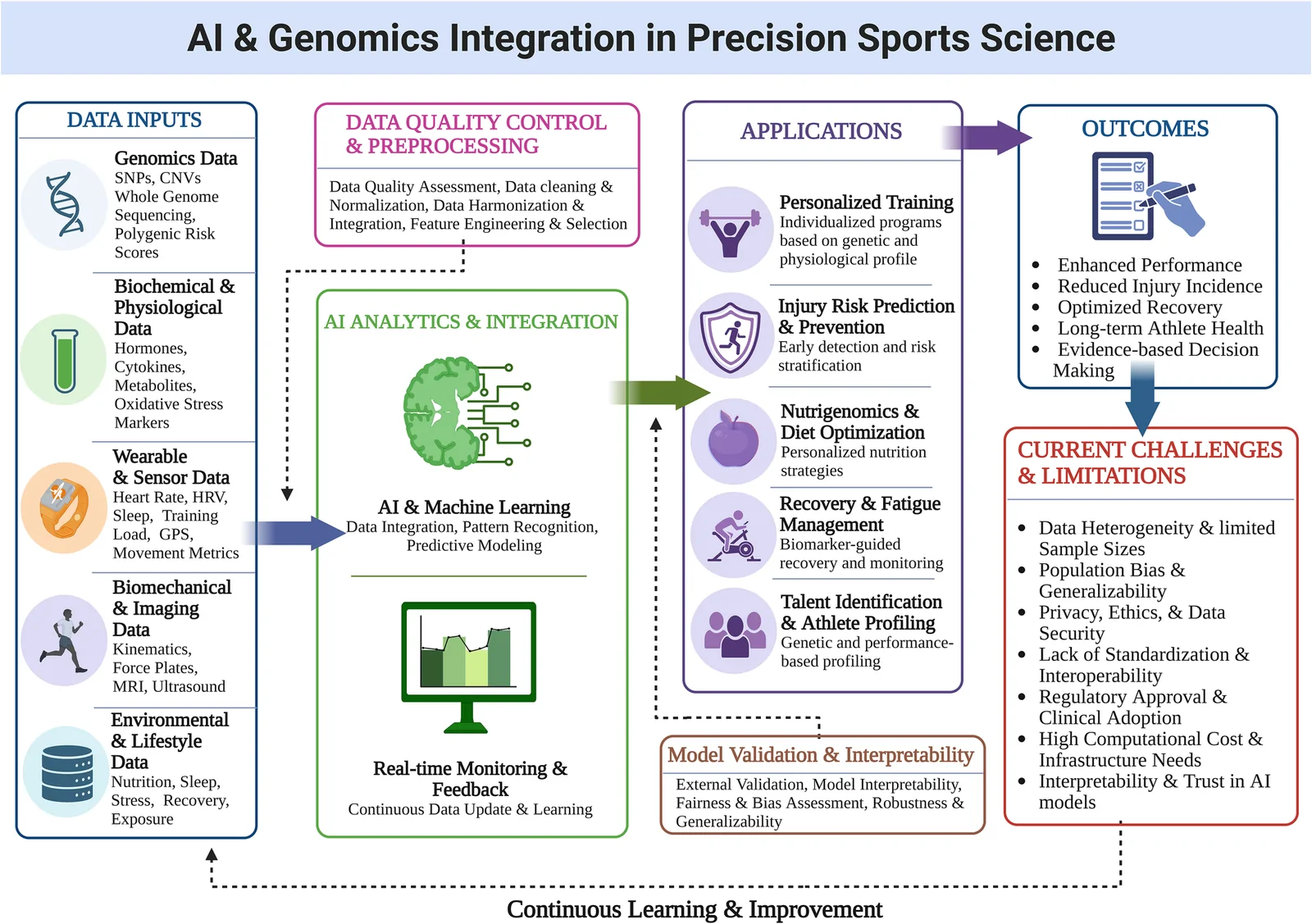
Conceptual framework of artificial intelligence and genomics integration in precision sports science. (*Created in BioRender. Patel, A. (2026)* https://BioRender.com/2ox5xys).

Sports science is changing as a result of the combination of genetics and artificial intelligence (AI), which offers unprecedented opportunities to improve athletic performance. Complex connections between genetic predisposition, training, and diet lead to elite athletic performance (1). However, significant interindividual heterogeneity resulting from standard training remains a persistent problem (2, 3). Because of genetic differences and gene-environment interactions, some athletes show notable adaptations while others have limited responses (4). The unique biological characteristics of every athlete are ignored by traditional approaches based on standardised protocols. This field is currently changing towards predictive, personalised, and preventive frameworks due to the development of high-throughput genomics, wearable sensors, and machine learning (5) (Figure 1). In this review, we operationally define “precision sports science” as an evidence-based framework that integrates multi-source biological and environmental data (genomic, physiological, biomechanical, psychological) with computational models to stratify athletes into risk-based or response-based subgroups, enabling targeted interventions that may improve performance or reduce injury incidence compared with standard-of-care protocols. We distinguish “precision” (subgroup-level stratification, which is emerging) from truly “personalised” (individual-level optimisation, which remains largely aspirational). However, substantial interindividual variation in adaptation and injury risk has largely been overlooked by traditional approaches (2–4, 6).

Genetic variation contributes to athletic performance, but the evidence remains complex and inconsistent. Research has progressed from candidate-gene studies to genome-wide association studies (GWAS) and polygenic approaches. A meta-analysis by İpekoğlu et al. (7) identified association between power performance and variants including *GALNTL6* rs558129, *NOS3* rs2070744, and *MCT1* rs1049434, while *ACTN3* R577X, *ACE* I/D, and *PPARA* polymorphisms remain among the most extensively investigated markers. However, athletic performance is highly polygenic, individual variants generally explain only a small portion of phenotypic variance, and many reported associations lack independent replication (7). The predominance of Caucasian cohorts further limits the generalisability of existing findings to underrepresented populations.

AI can handle these issues well. AI algorithms may combine multi-omics data, model nonlinear genetic variant interactions, and use real-time physiological inputs. Synthetic data reduces data scarcity, according to Cordeiro et al. (8). Using genetic SNPs, biomechanics, and training data from 142 runners (9), predicted injuries with an AUC of 0.784. Further, polygenic risk scores improve injury prediction exercise-responsive microRNAs (miR-206, miR-486, and miR-133a), and relate genetics to training intensity (1). Using multi-omics, Reitzner et al. (10) found molecular fingerprints that differentiate endurance and power athletes. However, these studies relied on internal cross-validation without temporal or geographic external validation; whether these models maintain accuracy when deployed on new teams or seasons remains unproven.

In the field of sports genomics, deep learning is a promising development. By combining a Genomic Athletic Predictor and uncertainty-aware prediction, Dong & Lv (11), presented a system that outperforms traditional models A neural network constructed by Kathuria et al. (12) successfully identified swimmers by genetics (12). Yet these deep learning approaches have been tested on fewer than 150 athletes and lack prospective validation in independent cohorts. Despite these developments, most research uses small, primarily Caucasian cohorts, which limits the generalizability of the findings (1). Also mentioned earlier, implementation is further complicated by ethical concerns regarding genetic data privacy (13).

This article presents a narrative review with systematic search elements, synthesising peer-reviewed evidence from 2014 to 2026 to address three specific questions: (i) What is the current strength of evidence linking specific genetic variants to athletic performance and injury risk, and what methodological limitations undermine these associations? (ii) How effectively do AI and machine learning algorithms integrate genomic, multi-omics, and physiological data to predict performance and injury outcomes, and do they demonstrate external validity? (iii) What ethical technical, and translational barrier must be overcome before precision sports science can move from research concept to applied practice?.

We discuss sports AI-genomics applications, challenges, and future prospects, including AI-driven performance analysis, genomic analysis (SNP-based and polygenic risk scoring), integrated predictive modelling (power vs. endurance classification and multi-omics integration), systems biology approaches (genotype-phenotype correlations and network-based pathway modelling), personalised training, and injury prediction. This review makes two original contributions: (i) it systemically evaluates whether genomic data adds incremental predictive value beyond conventional sports science variables using a value-of-information framework; and (ii) it integrates ethical, methodological, and technical limitations into a unified “Implementation Gap Analysis” rather than treating them as afterthoughts. This review synthesises recent original research to help researchers, coaches, trainers, physicians, and policymakers understand AI and genomics' current state, limitations, and future potential in improving athletic performance, preventing injuries, and improved personalised training.

## 2 Molecular and biochemical foundations of athletic performance

Complex interactions between molecular genetics, metabolic pathways, and adaptive cellular signalling systems underpin athletic performance. Recent study shows that *ACTN3*, *ACE*, and *PPARGC1A* gene variants affect metabolic capacity, cardiovascular efficiency, and muscle fibre composition. Genome-wide research showed more than 150 polymorphisms linked to power-related sports (14) The biochemical level determines performance by tightly controlling the energy system, which includes the glycolytic, phosphagen, and oxidative pathways. Enzymatic regulators and mitochondrial function also play a role, as they determine energy availability, resistance to tiredness, and recovery efficiency (15). New study highlights the importance of epigenetic modifications like DNA methylation and microRNA activity in modifying the expression of genes corresponds to training stimuli. This affects the flexibility and metabolic adaptation of skeletal muscles (16) Furthermore, integrative “omics” methodologies (genomics, metabolomics, proteomics) and biomarker profiling are enhancing precision sport science by facilitating personalized training and recovery plans based in biochemical and molecular profiles (6). These findings show that molecular, metabolic, and environmental variables affect athletic performance in a polygenic and dynamic manner.

### 2.1 Genetic and molecular determinants of athletic performance

#### 2.1.1 Key genes and polygenic traits

Sports genomics demonstrates that athletic performance is a highly polygenic characteristic affected by the cumulative impact of numerous genes that control muscle function, cardiovascular capability, and metabolic efficiency (17). *ACTN3*, *ACE*, *PPARGC1A*, and *NOS3* are among the most investigated genes for sports performance. Thus, *ACTN3* and *ACE* gene polymorphisms have been associated to sprinting, muscle strength, and aerobic endurance, which are crucial to team sports performance (18). The *ACTN3* gene is associated to strength and speed. The *ACTN3* R577X polymorphism changes muscle fiber composition and helps elite power athletes perform. *ACE* genes, particularly the I allele, are thought to manage blood pressure and endurance, improving metabolic efficiency and endurance sports performance (19). The R577X *ACTN3* gene polymorphism affects *α*-actinin-3 protein expression in type II (fast-twitch) muscle fibers. RR genotype athletes are more likely to excel in power and speed sports, while XX genotype athletes may excel in endurance sports (20). Another important polymorphism is ACE I/D, where the I allele improves endurance performance while the D allele improves anaerobic and strength performance (19). Genetic variations can alter player placement and training in team competitions. *PPARGC1A*, which regulates biogenesis of mitochondria and energy metabolism, and IL6, which regulates inflammatory response and recovery, reveal how genetics affect performance and injury susceptibility (21) (Table 1). Genetic associations reported in Table 1 represent statistical correlations from observational studies, not demonstrated casual relationships. Effect sizes are population averages and do not predict individual outcomes.

**Table 1:** Major genetic polymorphisms associated with athletic performance traits.

Gene & variant	Associated trait/Performance phenotype	Mechanism/Functional effect	Effect size/OR	Replication status & population limitations	References
*ACTN3* 577RR	Sprint/power performance, elite sprinting, strength	*α*-actinin-3 present in type II fast-twitch fibers; enhances explosive contraction	OR ∼1.48 (power)	Replicated in multiple cohorts; primarily European/Asian populations	(22, 23)
*ACTN3* 577XX	Endurance advantage in some populations (subtle)	α-actinin-3 absent; possible metabolic shift to oxidative fibers	Effect small (*d* < 0.3)	Inconsistent replication; population-dependent	(23, 24)
*ACE* II	Endurance performance, lower fatigue, better efficiency	Lower *ACE* activity → less angiotensin II → improved vasodilation & oxygen utilization	OR ∼1.54 (endurance)	Moderately replicated; European bias	(22, 25)
*ACE* DD	Higher power output, strength gain with training	Higher *ACE* activity → increased angiotensin II → possible muscle hypertrophy	OR ∼1.56 (power)	Moderately replicated; European bias	(22, 25)
*PPARGC1A* Gly/Gly	Increased likelihood of elite athlete status (Caucasians)	Transcriptional coactivator regulating mitochondrial biogenesis and oxidative metabolism	OR ∼1.3–1.8	Limited replication; Caucasian-specific	(26, 27)
*PPARGC1A* Ser/Ser	No significant association (Caucasians); lower likelihood of elite power status (Asians)	Reduced *PPARGC1A* activity → decreased mitochondrial density	Non-significant in most studies	Poor replication across ethnicities	(26, 27)
*CKMM* G allele	Higher baseline strength & response to resistance training	Muscle-specific creatine kinase; affects energy buffering in fast-twitch fibers	Explains ∼2%–3% variance	Few replication studies	(28, 29)
*VDR* B allele	Greater muscle strength, lean mass	Vitamin D receptor → influences muscle protein synthesis & calcium handling	Small effect (*d* < 0.4)	Inconsistent findings	(30, 31)
*MSTN* R allele	Increased muscle mass, elite power athlete status (rare)	Myostatin inhibitor variant; reduces negative regulation of muscle growth	Very rare variant	Case reports only; not replicated in GWAS	(32, 33)
*COL5A1* CC	Reduced risk of tendon/ligament injury; overrepresented in elite rugby athletes	Alters collagen V α1 chain → affects fibril assembly & tensile strength	OR ∼0.6–0.8 (protective)	Mixed replication; European bias	(34, 35)
*ADRB2* Arg/Arg	No significant effect on bronchodilation or performance (in cyclists with salbutamol)	*β*2-adrenergic receptor → bronchodilation & metabolic effects	Non-significant	Well-replicated null association	(36, 37)
*HIF1A* Pro/Pro	Improved endurance capacity	Hypoxia-inducible factor 1α → enhances erythropoiesis & glycolysis	Small effect	Limited replication	(38, 39)

OR = odds ratio; d = Cohen's d effect size. Most individual variants explain <1–2% of phenotypic variance. Approximately 80% of reported performance-associated SNPs have not been independently replicated. Effect sizes are population averages and do not predict individual outcomes.

#### 2.1.2 Muscle fiber composition and metabolic specialization

Skeletal muscle contains slow-twitch (type I) and fast-twitch (type II) fibers, supporing endurance and explosive movements, respectively (40). Type I fibers have high mitochondrial density, whereas type II fibers include include fast-oxidative (type IIa) and fast-glycolytic (type IIb/x) subtypes. These fibers express distinct myosin heavy chain (MHC) isoforms encoded by *Myh7*, *Myh2*, *Myh1*, and *Myh4, Myh4* for types I, IIa, IIx and IIb, respectively (41). During sprinting, energy production shifts rapidly from oxidative phosphorylation toward anaerobic glycolysis, increasing metabolic intermediates that support cellular energy metabolism (42, 43).

#### 2.1.3 Critical limitations of candidate gene studies

The candidate gene era (circa 2000–2015) produced many of the associations catalogued in Table 1, but this approach is now recognised as fundamentally limited for polygenic traits such as athletic performance. Four major limitations undermine the clinical utility of candidate gene findings:

##### 2.1.3.1 Small sample sizes and winner's curse

Most candidate gene studies enrolled fewer than 200 athletes. Statistical power calculations indicate that detecting an SNP explaining 1% of phenotypic variance with 80% power at *α* = 0.05 requires *n* = 2,200 participants; for case-control studies with odds ratios of 1.2, approximately 4,000 cases and 4,000 controls are needed (44) The vast majority of sports genomics studies fall orders of magnitude below these thresholds, producing inflated effect sizes that shrink upon replication.

##### 2.1.3.2 Population stratification and ethnic bias

Allele frequencies differ markedly across populations. For example, the *ACTN3* R577X R allele frequency is ∼80% in African populations, ∼50% in European, and ∼35% in East Asians. A study finding RR genotype enrichment in elite sprinters may reflect population ancestry rather than true athletic association if controls are not ancestry-matched (45).

##### 2.1.3.3 Limited explained variance

Even the most robust single-gene associations explain only a small fraction of phenotypic variance. The *ACTN3* R577X variant explains approximately 2%–3% muscle strength variance; ACE I/D explains <2% of endurance phenotypic variance. Because performance is polygenic, involving hundreds to thousands of variants each with minute effects, single-SNP testing cannot yield clinically useful predictions (17, 46).

##### 2.1.3.4 Lack of independent replication

A systematic evaluation found that approximately 80% of genetic markers purportedly linked to athletic performance have not been replicated in independent cohort (1). Replication failures are particularly common for collagen-related injury genes (*COL5A1, COL1A1*) and inflammatory modifiers, where initial positive findings often disappear in larger, better-controlled studies (47).

Consequently, we emphasise that current genetic tests derived from candidate gene studies cannot reliably predict individual athletic performance or injury risk. Their use of talent identification in minors is scientifically unjustified and ethically problematic (48).

### 2.2 Biochemical markers for athletes' performance, fatigue, and adaptation

Sports science literature has extensively explored how training load (TL) affects biochemical markers of physiological stress and recovery (49–51). Markers including creatine kinase (CK), C-reactive protein (CRP), and creatinine, have been associated with exercise-induced muscle injury and training load variations (52). Salivary immunoglobulin-A (s-IgA) and *α*-amylase (s-AA), indicate acute stress and have been used to monitor changes in training in soccer players (53–55) (Table 2).

**Table 2:** Biochemical biomarkers used in sports performance monitoring and recovery assessment.

Biomarker	Physiological significance	Application in sports science	AI integration status	Key observation	References
CK	Muscle membrane damage	Monitor muscle stress & recovery	Used in Rossi & Rodrigues 2025 ML model	High levels after 2–3 days mean slow recovery	(56–58)
LDH	Tissue damage marker	Track tissue injury	Limited AI integration to date	Takes 6–8 days to return to normal after marathon	(59, 60)
CRP	Acute inflammation	Assess systemic inflammation	Emerging in injury prediction models	Stays high for 8 days after marathon if inflamed	(59, 61)
Lactate	Anaerobic glycolysis end product	Determine intensity & fitness	Widely used in real-time wearable AI	Clears within 1–2 h in fit athletes	(62, 63)
Ammonia	ATP degradation product	Monitor fatigue	Limited integration	Lower during competition means better adaptation	(63, 64)
Hypoxanthine	ATP depletion marker	Monitor training load	Limited integration	Lowest during competition means good adaptation	(63, 65)
Testosterone	Anabolic hormone	Assess anabolic capacity	Used in Mohsin et al. (67) AI model	Low levels mean overtraining; normal means recovered	(56, 66, 67)
Cortisol	Catabolic stress hormone	Evaluate strain	Used in Mohsin et al. (67) AI model	Slow to return to normal means poor recovery	(56, 67, 68)
T/C Ratio	Anabolic-catabolic balance	Track fatigue risk	Used in ensemble ML models	Low ratio means body breaking down; normal means adapting	(69, 70)
Ferritin	Iron storage protein	Screen for iron deficiency	Limited AI integration	Below 75 means low iron stores	(71, 72)
s-IgA	Mucosal immunity	Predict infection risk	Used in HRV + load composite models	Low levels = 2–3x higher risk of cold/flu	(73)
Uric Acid	Purine metabolism/oxidative stress	Assess metabolic disruption	Limited integration	Still high after 24 h means body not recovered	(56, 74)
IL-6	Inflammatory cytokine	Assess exercise-induced inflammation	Emerging in multi-omics AI models	High after exercise; lower with good omega-3 status	(75, 76)
Omega-3 Index	Membrane inflammation resolution	Evaluate nutrition status	Limited direct AI integration	Higher index = less muscle damage and inflammation	(75, 77)
hs-Troponin T	Cardiac stress marker	Monitor heart strain	Used in cardiac monitoring AI	Goes up after marathon; returns to normal by 4 days	(59, 78)

CK, creatine kinase; LDH, Lactate dehydrogenase; CRP, C-reactive protein; T/C, testosterone/cortisol; s-IgA, salivary immunoglobulin-A; IL-6, interleukin-6; hs-Troponin T, high-sensitivity troponin T. AI integration status indicates current use as features in published machine learning models.

From an AI perspective, these biomarkers represent potential input features for predictive models, yet their integration into sports AI remains limited. Most current injury prediction algorithms rely primarily on non-invasive wearable data (GPS, accelerometery, HRV) because biomarker collection requires invasive sampling and introduces latency (23–72 h for CK). Future multi-modal AI systems may incorporate biomarkers as high-value, low-frequency features, but prospective studies demonstrating incremental predictive utility beyond wearable data are scarce (6, 79).

Energy metabolism markers-including blood lactate, glycogen, glucose, free fatty acids, and enzymes such as citrate synthase-measure substrate usage, mitochondrial efficiency, and metabolic adaptation (80, 81). These metrics help determine player fatigue, recuperation, and training efficacy (82).

Biomarker latency and invasiveness explain why current AI injury-prediction models rely primarily on wearable data, and that biomarker integration remains a future challenge.

### 2.3 Epigenetics and gene–environment interactions in performance

Recent research emphasises physical exercise as an environmental modulator that alters epigenetics, enhancing gene expression and controlling physiological processes (16, 83, 84). DNA methylation, RNA methylation, histone modifications, and non-coding RNAs modify gene expression without altering DNA sequence. Physical activity causes epigenetic changes that improve physiological processes and lower disease risks (85). Exercise-induced hypomethylation of genes associated with glucose transport (e.g., GLUT4) (16) and mitochondrial regulation (e.g., PGC-1*α*) augments endurance adaptations and metabolic efficiency (86).

However, casual attribution is challenging: observational epigenetic studies cannot distinguish exercise-induced methylation changes from pre-existing differences that predispose individual to exercise participation. Randomised controlled trials with epigenetic endpoints in athletes remain rare (16).

Athletes with similar genetic backgrounds may have variable performance outcomes based on lifestyle, altitude exposure, food, sleep quality, and physiological stress (17). Gene-environment interactions nutritional factors modulate epigenetic responses associated with performance and recovery (87).

Comparing Sections 2.1–2.3 reveals a consistent pattern: molecular plausibility exceeds clinical utility. While *ACTN3*, *ACE*, and *PPARGC1A* have robust biological rationales, the effect sizes are small, replication is inconsistent, and predictive utility for individual athletes is negligible. Biochemical markers (CK, cortisol, s-IgA) correlate with training load but lack specificity for injury prediction. Epigenetic changes are dynamic and reversible, making them poor static predictors. For AI integration, these biological data streams are best treated as supplementary features within multi-modal models rather than standalone predictive tools.

## 3 Artificial intelligence in sports science

AI is changing various domains, including sports science. AI tools are transforming athlete training, rehabilitation, and performance by analysing vast datasets and identifying patterns to generate predictive insights (88, 89). AI can improve biomechanics analysis, rehabilitation, and sports injury prevention (90). Empirical and data-driven AI interventions can improve sports decision-making (91).

Machine learning algorithms can now detect fatigue, injury susceptibility, and poor performance by analysing training loads, biomechanical patterns, and physiological responses (92). Movement symmetry, functional recovery, and individualised treatment are also being assessed using neural networks and deep learning models (93).

### 3.1 Overview of artificial intelligence techniques in sports analytics

#### 3.1.1 Machine learning and deep learning methodologies

Complex OMICS, biomechanical, and physiological, data may be analysed using advanced machine learning algorithms to accurately predict injury risk (94). Computer algorithms handle raw high-dimensional data, detect non-linear relationships, and improve through iterative learning, surpassing conventional statistical methods (95). Recently published bibliometric studies show exponential growth in exercise science AI applications. Research is transitioning to prediction and prevention of injury (96). Traditional injury prevention measures are helpful, but they cannot fully leverage the wealth of data accessible through modern monitoring systems and OMICS technologies (6).

In exercise medicine, deep learning and multilayer neural networks have altered biomechanical analysis. These advanced algorithms can analyse raw kinematic and kinetics data to find minor movement patterns linked to injury risk, frequently discovering aberrations that even expert clinicians overlook (97). Recently, frequent neural networks with long short-term memory (LSTM) architecture have been shown to predict essential gait events such minimum foot clearance (MFC), which is crucial to tripping risk (98). These algorithms can identify potentially dangerous movement patterns using toe-off kinematics with mean absolute errors as low as 0.07 s in MFC time (99).

Outside labs, wearable sensors and smartphone-based assessments employ deep learning. Computer vision algorithms like OpenPifPaf can estimate joint kinematics without biomarkers with a root mean square errors of fewer than 6 degrees, comparable to gold-standard marker-based methods. Lambricht et al. (100) found that these assessments are valid when athletes wear typical running clothing, suggesting their usage outside of labs. These methods democratise biomechanical research by allowing continuous monitoring throughout training and competition.

#### 3.1.2 Predictive modeling approaches

AI-driven predictive analytics and athlete monitoring avoid team injuries. Machine learning algorithms can improve training periodization to decrease injury risk and maximise performance by analysing training loads, recovery measures, competition schedules, environment factors, and team-wide OMICS profiles. In rugby, massive datasets of head impact footage and clinical outcomes have helped construct concussion risk prediction systems (101).

Using AI to prevent team injuries is tactical and strategic. Coaching can avoid injuries without sacrificing performance using machine learning algorithms to recognise game conditions, positional tasks, and strategies that increase risk (102). These investigations utilising genetic and proteomic data can better assess whether athletes need additional preventative measures in competitive situations based on their risk profiles (6).

### 3.2 Data sources for artificial intelligence in sports science

**Figure 2:**
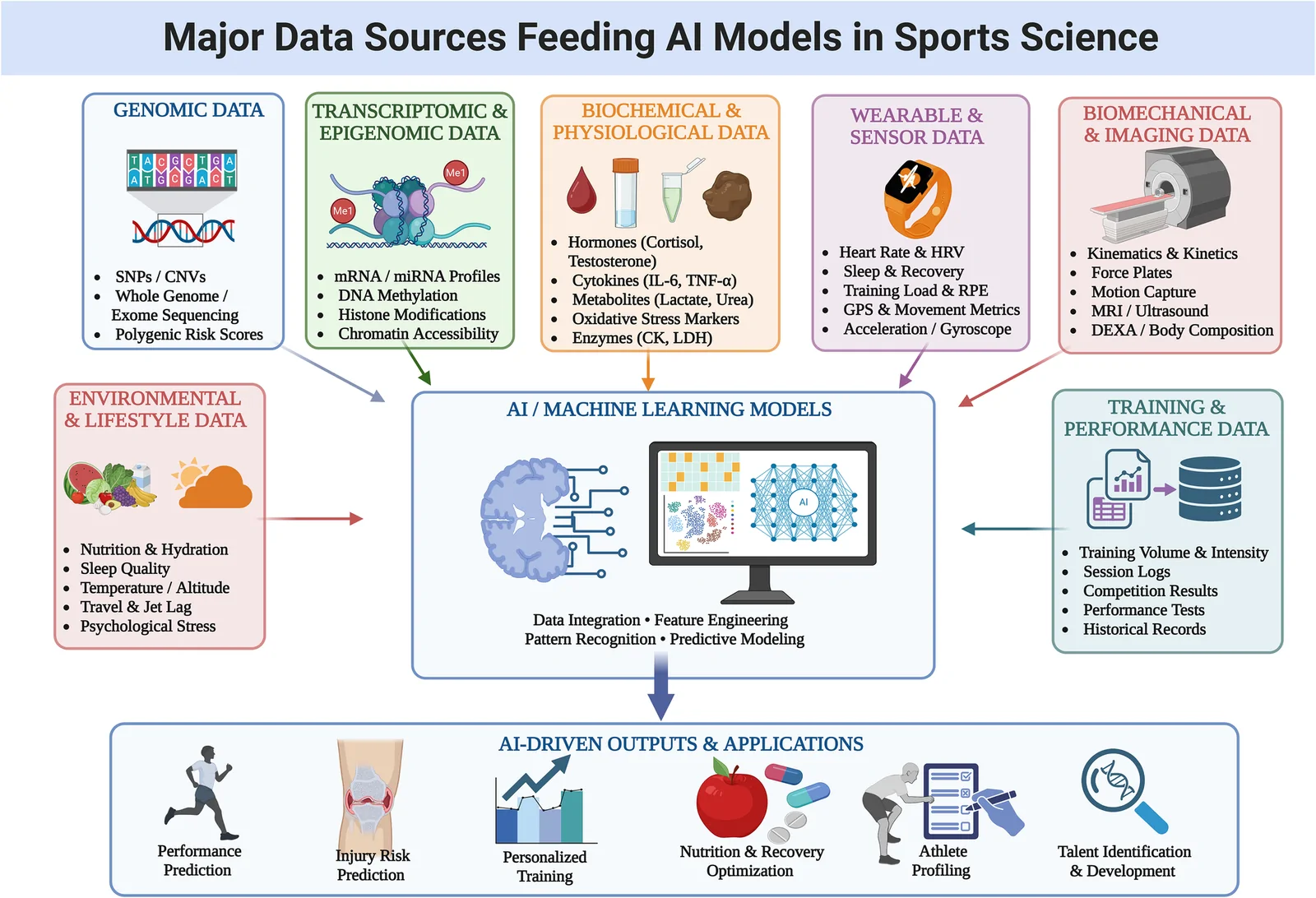
Major data sources feeding AI models in sports science. (*Created in BioRender. Patel, A. (2026)* https://BioRender.com/2ox5xys).

AI and ML algorithms can analyse genetic predispositions, protein biomarkers, metabolic pathways, gene expression patterns, and real-time physiological data from wearable sensors (103) (Figure 2).

#### 3.2.1 Genomic and multi-omics data

Genomic data supports precision sports medicine by revealing genetic factors affecting athletic performance, injury susceptibility, and recovery (104). Genome-wide association studies found athletic SNPs. These include the *ACE* I/D polymorphism, which affects cardiovascular efficiency, the *ACTN3* R577X variant, which affects muscle fibre structure and sprint performance, and the *COL5A1* variant, which affects connective tissue integrity and ligament injury risk (1, 105). The importance of genomic profiling is demonstrated by the fact that genetic factors account for around 66% of the diversity in athletic ability and status (104). However, as detailed in Section 2.1.3, the predictive value of this individual variant is limited, and their inclusion in AI models must be treated as exploratory features requiring rigorous validation.

Besides genetic markers, multi-omics includes transcriptomics, proteomics, and metabolomics (6, 104). The molecular landscape of an athlete throughout training, performance, and recovery is dynamically represented by omics technology. Metabolomics can show elite athletes’ energy utilisation, muscle regeneration, and fatigue tolerance. Polygenic risk scores and prediction models can predict performance and injury risk using AI analytics and molecular data, though current polygenic models in sports lack external validation and explain only modest proportions of variance (1, 105).

#### 3.2.2 Wearable and sensor-based physiological data

AI applications in sports science benefit from real-time physiological data collection via wearable sensors (79, 106). Contemporary wearable technology, including inertial measurement units (IMUs), GPS trackers, and heart rate monitors, and near-infrared spectroscopy (NIRS) sensors, incessantly gather high-frequency data regarding athletes' movement, cardiovascular performance, and tissue oxygenation (79, 105). These sensors provide critical data like speed, distance, acceleration and deceleration patterns, heart rate variability (HRV), sleep quality metrics, and training load intensity (79, 106).

Integrating data from wearables with AI analytics enables real-time observation of an athlete's readiness, identification of non-functional overreaching, and early detection of potential injury risks (79, 105). The temporal sequences produced by wearable sensors are well-processed by deep learning architectures, especially recurrent neural networks (RNNs) and long short-term memory (LSTM) networks. This facilitates the prediction of injury risks and performance results based on cumulative training load patterns. A recent narrative study on deep learning applications in sports performance analysis found that combining visual, physiological, and inertial sensor data into a single model greatly improves accuracy and strengthens the dependability of real-time feedback systems (106).

#### 3.2.3 Biochemical and clinical performance indicators

Clinical biomarkers from blood, saliva, and urine objectively analyse an athlete's physiological status, recovery progress, and injury healing. Common biological data sources include blood tests, metabolic panels, hormonal assays, muscle injury markers, and inflammatory cytokines (TNF-α, IL-6). These indicators reflect the body's systemic response to training stress before clinical signs, making them helpful for AI-driven injury risk assessment (6, 79).

Pharmacogenomic data, which demonstrates the influence of genetic variations on individual responses to frequently used medications in sports medicine, such as cardiovascular agents (SLCO1B1 andCYP2C19), opioids (CYP2D6), and non-steroidal anti-inflammatory drugs (CYP2C9) variations, further clarifies this category. The AI system may use this data to provide the optimal drug regimens that minimise side effects and expedite safe return-to-play timeframes, though clinical implementation remains at the research stage (104).

#### 3.2.4 Biomechanical and imaging data

Motion capture systems, force plates, and diagnostic imaging give high-resolution biomechanical data on movement quality, joint kinematics, and tissue structure (79, 106). Force plates measure countermovement jumps characteristics, asymmetries, and peak power production, while three-dimensional motion analysis measures joint angles, ground response forces, and segment accelerations during sports. Musculoskeletal ultrasonography and magnetic architecture metrics including cross-sectional area, fascicle length, and pennation angle, help identify subclinical tissue damage (79).

Convolutional Neural Networks (CNN)can automatically recognise actions, classify movements, and identify biomechanical injury risk factors like anterior cruciate ligament rupture in visual and imaging data (6, 106). When biomechanical data is integrated with other data modalities, powerful prediction models can identify athletes at risk of injury before tissue damage (6).

### 3.3 Applications of artificial intelligence in athletic performance analysis

#### 3.3.1 Pattern recognition and data mining in athlete datasets

The digitisation of athletic training has created diverse, high-dimensional datasets of biometrics (heart rate, heart rate variability), biomechanical signals (joint angles, ground reaction forces), neuromuscular metrics (electromyography), and spatiotemporal tracking data. Conventional statistical approaches often miss large latent patterns in complex data, artificial intelligence, notably using clustering and dimensionality reduction are very helpful to finds hidden patterns (107, 108).

Adeyemo et al. (109) thoroughly compared three pattern mining algorithms: l-length Closed Contiguous Sequential Pattern Mining (LCCspm), Longest Common Subsequence (LCS), and AprioriClose, using movement data from 319 professional rugby league games. The LCCspm approach, which is designed to identify successive movement sequences, obtained a classification accuracy of 91.02% when paired with a multilayer perceptron neural network to differentiate between positional categories (e.g., hookers vs. wingers). The authors conclusively demonstrated that closed continuous patterns perform better in athlete profiling than non-consecutive sequences, indicating that the temporal ordering of movements contains crucial discriminating information.

K-means clustering was utilised on 31 elite young soccer players over two seasons by de Haan et al. (110). Clustering study employing sprint, intermittent endurance, and match-derived running parameters (total distance, high-intensity running distance, accelerations) showed three player phenotypes. Sprinting and endurance associated with match-specific running. To reduce overuse injuries, this subgroup enabled practitioners to identify players who needed alternate strategic roles or training loads. Pattern detection algorithms provide data-driven, personalised athletic development in large workout plans.

#### 3.3.2 Development of predictive models for performance optimization

Supervised and reinforcement learning approaches simplify performance prediction in training and competitive contexts, together with descriptive pattern recognition. These models help periodize real-time training and create competitive strategies.

In a comprehensive integrative study, researchers created a hybrid machine learning framework that incorporates physiological and psychological aspects. Using data from 480 athletes across sports, the final ensemble model predicted performance outcomes with 90% accuracy (R^2^ = 0.90) using gradient boosting machines and deep neural networks. Features importance analysis shows that functional movement screening scores (13.7% relative importance), athlete dedication (11.5%), and maximal acceleration capability (10.2%) are the most important predictors, highlighting the complexity of athletic performance (111).

Van Deuren et al. (112) tested XGBoost, random forests, and counterfactual recurrent networks to predict professional football players' session rating of perceived exertion (sRPE) for training load management. The XGBoost model improved prediction accuracy (RMSE = 1.262, R^2^ = 0.74), with total session distance, high-intensity running distance, and player age as key factors influencing perceived effort. The study's novel prescriptive counterfactual recurrent network predicted future RPE trajectories under various hypothetical training regimens with slightly lower predictive accuracy (RMSE = 1.379). Causal machine learning provides counterfactual insights for personalised training suggestions.

However, these studies relied on internal cross-validation without temporal or geographic external validation; whether these models maintain accuracy when deployed on new teams or seasons remains unproven.

Novel reinforcement learning (RL) architectures revolutionise dynamic, sequential training optimisation decision-making. Zhang et al. (113) developed a Deep Q-Network (DQN) architecture for track and field athletes' adaptive training loads. The reinforcement learning agent used a digital twin methodology based on longitudinal physiological data (HRV, sleep quality, acute:chronic workload ratio) from 25 athletes throughout a competitive season to improve short-term performance and long-term health. The optimised technique outperformed static periodization models and threshold-based heuristics in dynamically reducing injury risk and retaining athlete performance within personalised optimum limits (16). Zhou & Zhou (114), used a Markov decision process framework on the publicly available FitRec dataset to improve predicted performance metrics by 15% and reduce simulated training overload by 30% compared to baseline scheduling algorithms. Yet these RL approaches have been tested on fewer than 30 athletes and lack prospective validation in independent cohorts.

In order to anticipate football action sequences based on player and ball location data in team sports that need spatiotemporal sequence prediction, Zareba et al. (115) evaluated gradient boosting methods (CatBoost, LightGBM, XGBoost) with recurrent neural network architectures. When predicting goal-scoring opportunities many acts ahead of time, ensemble boosting models achieved an F1 score of 0.707 and a precision-recall area under the curve (PR AUC) of 0.734. This predictive methodology differs from conventional reactive analytics by offering crucial lead time for quick tactical adjustments.

These predictive and prescriptive AI frameworks together transform sports analytics from historical performance reporting to anticipatory, personalised performance improvement. However, there is still more research to be done on these models' applicability to various sports, competition levels, and environmental circumstances (116).

#### 3.3.3 Explainable artificial intelligence in sports analytics

Predictive performance alone is insufficient for clinical translation; models must be interpretable to support practitioner decision-making. Explainable AI (XAI) methods-such as SHAP (Shapley Additive exPlanations) values, LIME (Local Interpretable Model-agnostic Explanations), and attention mechanisms in deep learning-can elucidate why an algorithm assigns a particular risk score to an athlete. For example, an interpretable model might state: “This athlete is classified as high injury risk because the combination of *COL5A1* rs12722 risk allele, acute:chronic workload ratio >1.5, and HRV-derived sleep disturbance score exceeds the population threshold.” Currently, fewer than 10% of sports AI studies report interpretability metrics, representing a major translational barrier. Without XAI, coaches and clinicians cannot trust, audit, or act upon algorithmic recommendations (94, 95).

### 3.4 Reporting standards and quality frameworks for AI in sports science

To improve reproducibility and reduce publication bias, sports AI research should adhere to emerging reporting standards. CONSORT-AI and SPIRIT-AI extensions provide guidance for randomised trials involving AI interventions; TRIPOD-AI (currently in development) will standardise prediction model reporting; and the CLAIM-AI checklist (Checklist for AI in Medicine) offers criteria for study quality evaluation. Pre-registration of sports AI models on platforms such as OSF or ClinicalTrials.gov would further reduce selective reporting.

At present, none of the AI-genomics studies reviewed in Table 3 were pre-registered, and only three reported external validation, underscoring the need for methodological reform before clinical deployment.

**Table 3:** Artificial intelligence techniques applied in sports genomics research.

AI technique	Application in sports science	Dataset type	Outcome/Performance	Evidence level & validation	Key limitations	References
Random Forest	Injury prediction	Biomechanical and wearable sensor data	Improved injury classification accuracy	Level III; Internal CV only	Small cohorts; no external validation	(58, 127)
XGBoost	Training load prediction	GPS and physiological data	Accurate prediction of perceived exertion	Level III; Internal CV only	Single-team studies; limited generalizability	(112, 128)
Deep Neural Networks	Athlete classification	Genomic and physiological data	Accurate swimmer performance categorization	Level IV; Small cohort (*n* < 150)	No prospective validation; Caucasian bias	(12, 129)
Convolutional Neural Networks (CNNs)	Movement and injury analysis	Imaging and motion capture data	Detection of biomechanical injury risk factors	Level III; Internal CV	High computational cost; limited interpretability	(106, 130)
Recurrent Neural Networks (RNNs)	Temporal performance prediction	Wearable sensor sequences	Enhanced fatigue and recovery prediction	Level III; Internal CV	Data leakage risk; sequence dependency	(98, 131)
Long Short-Term Memory (LSTM)	Gait and motion analysis	Kinematic datasets	Precise gait event prediction	Level III; Internal CV	Limited to lab conditions; small samples	(99, 132)
Reinforcement Learning	Adaptive training optimization	Longitudinal athlete monitoring	Dynamic load adjustment and injury prevention	Level IV; *n* = 25 athletes	No independent replication; overfitting risk	(113, 133)
Polygenic Risk Modeling	Injury susceptibility prediction	SNP and multi-omics data	Improved ACL and muscle injury prediction	Level IV; No external validation	Small effect sizes; population stratification	(1, 6)
Clustering Algorithms (K-means)	Athlete phenotype classification	Match and physical performance data	Identification of athlete performance profiles	Level III; Descriptive	Arbitrary cluster number; validation unclear	(110, 134)
Gradient Boosting Models	Goal-scoring opportunity prediction	Spatiotemporal team-sport data	High predictive performance in football analytics	Level III; Internal CV	Position-specific bias; temporal leakage	(115, 135)

CV, cross-validation; Evidence Level: III, retrospective cohort or internal cross-validation; IV, case-control or proof of concept. None of the listed studies used pre-registered analysis plans or independent external validation cohorts. All models require prospective, multi-site validation before clinical deployment.

## 4 Integration of artificial intelligence and genomics in sports

### 4.1 Artificial intelligence-based analysis of genomic data in athletes

Athletes' genetic data has been analysed using machine learning and deep learning AI. In sports genomics, artificial intelligence models are being utilised to go beyond single gene or single nucleotide polymorphism study to help comprehend how genetic variation combinations affect training, injury susceptibility, and performance (6).

However, the integration of genomic and AI methodologies remains predominantly exploratory, with most studies using small, homogeneous cohorts and lacking external validation.

#### 4.1.1 SNP-based analysis and polygenic risk scoring

Highly polygenic athletic performance genetics include several SNPs with minor to moderate effects (19). Univariate association studies miss these genetic variables' intricate interactions, but whether machine learning algorithms genuinely capture meaningful nonlinear interactions-rather than fitting noise in small samples-remains debated.

Comprehensive sports genetics update from 2023 found 251 DNA variants associated with athletic status, of which 128 were verified in at least two investigations. While *ACTN3* rs1815739 (R577X) and *AMPD1* rs17602729 are linked to power, *PPARGC1A* rs8192678 (Gly482Ser), *PPARA* rs4253778, and *HFE* rs1799945 are the most promising markers linked to endurance. The results came from genome-wide association studies (GWAS), candidate gene studies, and meta-analyses that incorporated large-scale initiatives like the UK Biobank (17). Verification in two studies is a weak standard; independent replication in large, ancestrally diverse cohorts with standardised phenotyping is required before clinical utility can be claimed.

Using a case-control approach, Guilherme et al. (117) investigated genetic determinants of elite sprinter status. The study demonstrated the polygenic nature of power-based athletic performance by finding genetic variants associated with brisk walking that were also common among great sprinters. The authors employed a comprehensive genotype score technique that creates a composite score by combining the effects of many SNPs associated to performance.

Varillas-Delgado et al. (118) conducted genotyping of polymorphisms in genes associated with iron metabolism (*HFE*, *PGC1a*, *AMPD1*), cardiorespiratory fitness (*ACE*, *BDKRB2*, *ADRA2A*, *ADRB2*, *NOS3*,), and muscle injury susceptibility (*ACE*, *ACTN3*, *AMPD1*, *MLCK*, *CKM*) in elite endurance athletes for injury prediction. A subsequent investigation by the Varillas-Delgado et al. (119)analyzed *MLCK*, *AMPD1* rs17602729, *ACE* rs4646994, *ACTN3* rs1815739, and *CKM* rs8111989 polymorphisms in 100 top endurance athletes (*n* = 50 male and 50 female) utilizing real-time PCR, establishing a foundation for genetic profiling in injury risk evaluation.

Ahmetov et al. (46) conducted a thorough study of sports genomics, concluding that hundreds or possibly thousands of DNA polymorphisms are necessary for accurate predictions of athletic injury risk and performance. The authors highlighted that, although potential genetic markers have been identified, such as *COL5A1* rs12722 for soft-tissue injuries and *PPARG* rs1801282 for strength-elite performance, cannot currently be predicted with precision using genetic analysis alone.

Critically, these studies illustrate the divergence in genotyping panels and statistical threshold across laboratories, which produces inconsistent polygenic scores and undermines cross-study comparability.

#### 4.1.2 Feature selection, dimensionality reduction, and model optimization

The “curse of dimensionality” is a fundamental difficulty in the investigation of genomic and performance data. The number of features often exceeds the number of athlete samples, causing overfitting and poor generalizability. Artificial intelligence-based feature selection and dimensionality reduction are essential for developing parsimonious, interpretable models, and identifying physiologically or biomechanically important variables. In sports genomics, where sample sizes are typically 50–200 athletes and features counts can exceed 50,000 SNPs or 1,000 metabolites, the *P*>>N problem is acute. Without proper regularisation, independent validation, and prespecified analysis plans, reported accuracies are likely inflated.

Baldominos et al. (120) examined and compared several feature selection techniques using evolutionary algorithms to identify physical activity. The PAMAP2 dataset, a publicly available repository in the UCI Machine Learning Repository, was used to show that genetic algorithm-based feature selection reduced dimensions by almost 50% and achieved an average classification accuracy of 97.45%, exceeding previous literature results. Dimensionality reduction accelerated categorisation and lowered wearable sensor system energy and computational expenses.

Bouvet et al. (121) introduced a model-based dimension selection method for clustering multivariate functional data from inertial measurement units in swimming performance analysis. A sparse analysis of component distribution makes clustering easier by revealing key dimensions. With front-crawl swimmers, the recommended strategy consolidated kinematic stroke variability tied to swimming technical skills and made biomechanical sprint performance improvements easier to discover. The researchers discovered that selecting pertinent dimensions during clustering is essential since the structure of a swimming pattern determines how tough it is to replicate.

Vanumu et al. (122) created a machine learning framework for sports analytics model hyperparameter optimisation to handle data-scarce regression issues. They used combined hyperparameter optimisation and SMOGN (Synthetic Minority Over-Sampling Technique for Regression with Gaussian Noise). In a cricket performance prediction case study, the authors showed how systematic model parameter optimisation enhances predicted accuracy with minimal athletic performance data.

To enhance athlete speed training, an adaptive hierarchical evolutionary algorithm was devised. Using hierarchical genetic chromosomal encoding, this method optimises neural network architecture and weight threshold parameters while optimising solution weights. With adaptive hybridization and mutation rates improving genetic efficiency and preventing premature convergence, this approach demonstrated better and more reliable weight training results than basic genetic algorithms. The algorithm has been applied to improve speed training approaches for different sports and personal characteristics, meeting the need for customised training methods (123).

While these technical advances are promising, they were validated on public datasets (PAMAP2, UCI Repository) rather than elite athlete cohorts, and their transferability to high-performance sports remain uncertain.

### 4.2 Predictive modeling of athletic performance using integrated AI–genomics approaches

#### 4.2.1 Classification of athletes into performance categories (power vs. endurance)

**Figure 3:**
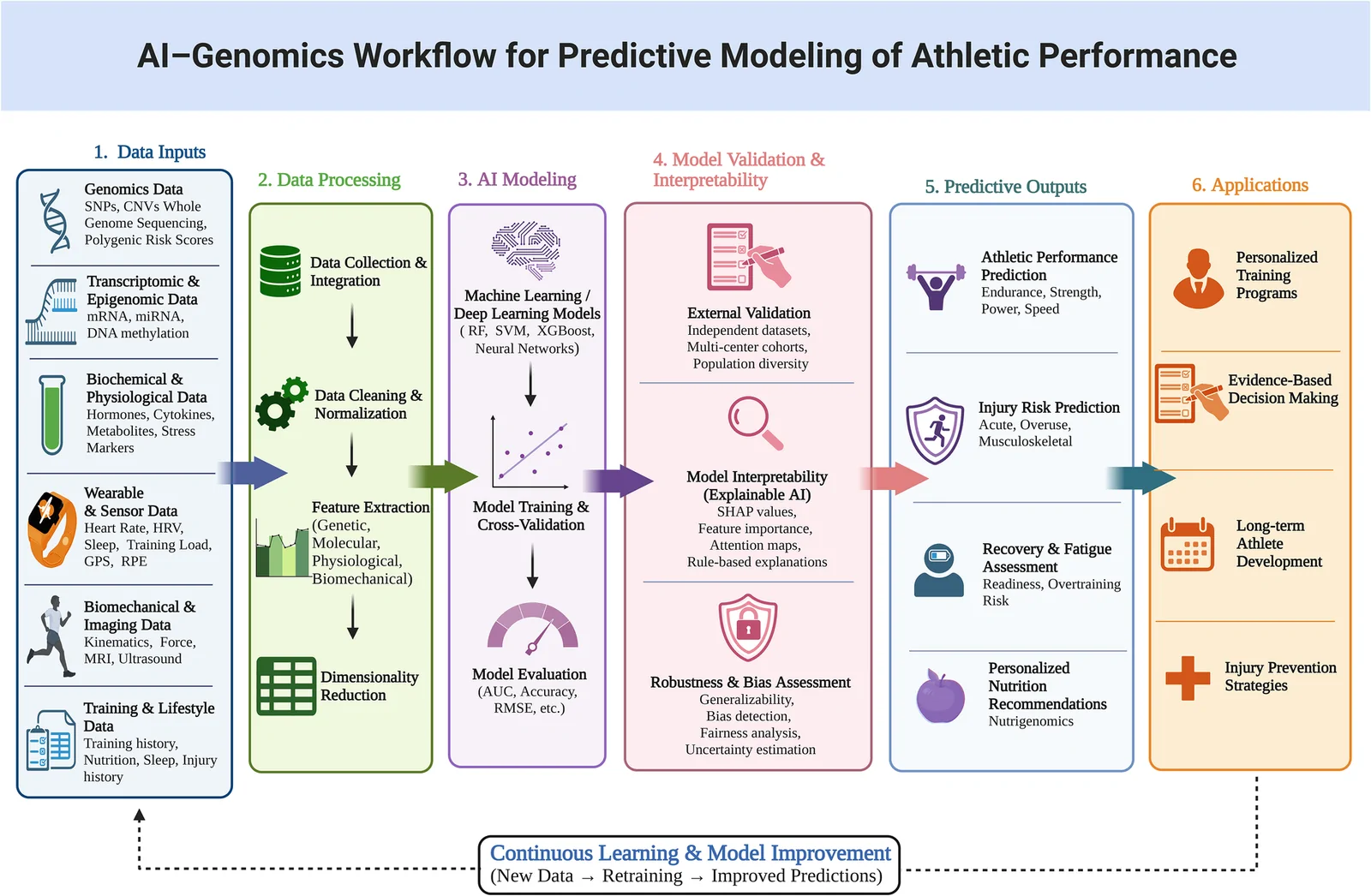
AI–genomics workflow for predictive modeling of athletic performance. (*Created in BioRender. Patel, A. (2026)* https://BioRender.com/2ox5xys).

One of the main uses of integrated AI-genomics techniques is the categorisation of athletes into different performance phenotypes, particularly power vs. endurance. Machine learning algorithms offer substantial advantages over traditional univariate statistical methods for distinguishing performance categories, given the polygenic nature of sports performance, which is defined by multiple SNPs with negligible individual effects (124) **(**Figure 3**)**. However, current classification accuracies (70%–80%) are only marginally better than chance and do not approach the reliability required for talent identification or training prescription.

Yang et al. (125) conducted a study on Chinese athletes' polygenic characteristics and peak power performance. 103 excellent Han Chinese athletes were studied in power (sprinters, throwers, jumpers) and endurance (middle and long-distance runners) categories. The scientists used logistic regression classifiers to predict talent identification utilising SNPs from potential genes including *ACE* rs4646994 (I/D), *ACTN3* rs1815739 (R577X), *PPARGC1A* rs8192678 (Gly482Ser), and *ADRB3* rs4994. The *ACTN3* R577X polymorphism was strongly correlated, with 70.8% of power athletes having the RR genotype and 53.5% of endurance athletes (*p* = 0.005). The study found comparable results in a different cohort of 125 top athletes, resulting in nomograms for successful talent recognition in both teenage and elite sports groups.

Pranckeviciene et al. (126) computed genotype scores that more accurately represented power and endurance traits in 180 elite Lithuanian athletes using logistic regression classifiers. Using four established markers—*ACE* rs1799752 (I/D), *ACTN3* rs1815739 (R577X), *AMPD1* rs17602729 (C34T), and the new marker *MB* rs7293 (myoglobin), the study generated a total genotype score (TGS) profile for endurance-mixed-power performance. Using data-driven TGS modelling, the scientists classified athletes by sport type (power vs. endurance) and found substantial sex-specific TGS distributions. The study made its computational tools available for free, allowing practitioners to utilise data-driven polygenic profiling instead of genotype ratings from literature.

Kathuria et al. (12) improved elite swimming performance prediction with deep learning, DNA markers, performance analytics, and physiological biometrics. Genetic markers, physiological data, and biomechanical traits were used to build a neural network model to classify swimmers by genetic predisposition and athletic potential. The model's high classification accuracy shows a strong relationship between genetic markers, physiological features, and competitive swimming. AI-driven analytics improves talent discovery, personalised training, and injury prevention, but more dataset diversity and epigenetic research are needed (Table 3).

Comparing Yang et al. (125) (Chinese cohort, logistic regression, *n* = 103) vs. Pranckeviciene et al. (126) (Lithuanian cohort, TGS modelling, *n* = 103) vs. Kathuria et al. (12) (deep learning, *n* < 50), highlighting how divergent genotyping panels, statistical thresholds, and ethnic backgrounds produce inconsistent classification accuracies (70%–80%) that are insufficient for clinical use.

#### 4.2.2 Integration of multi-omics datasets

Multi-omics datasets revolutionise sports research by shifting from single marker studies to molecular profiling of athletic performance. This approach, known as “sportomics,” combines transcriptomics, proteomics, metabolomics, genomics, and epigenomics to identify all-encompassing metabolic reactions in competitive real-time situations (124). However, the “curse of dimensionality” means that even in major research, the number of molecular features (P) often exceeds the number of participants (N), resulting in misleading connections when traditional statistical methods are used (1).

##### 4.2.2.1 The PhenoMol framework

To solve this dimensionality problem, Alizadeh et al. (136) created PhenoMol, which integrates large phenotypic data with multi-omic dimensionality reduction using graph theory limited by previous biological knowledge. The longitudinal multi-omics study collected 50,057 unique molecular measures from 86 highly active US Military Academy cadets, including 16,318 mRNA transcripts, 139 untargeted proteins, 571 untargeted metabolites, 16,544 gene regions, 16,415 gene promoters (DNA methylation), targeted metabolic and cytokine panels, and hematological and immunophenotypic measures. PhenoMol's fundamental innovation is a development of biologically informed “expression circuits”, groups of molecules that work together to separate high- and low-performing subgroups instead of molecular markers. PhenoMol predicted top physical performance better than regression models without network-based dimensionality reduction using an ACFT score threshold of 540 to distinguish higher- and lower-performers. Open software allows future study on health, performance, and disease outcomes in small and large populations. Despite its sophistication, the study enrolled only 86 participants far below the statistical power needed to detect modest genetic effects reliably and was restricted to a young, predominantly male, military population, limiting generalizability to civilian elite athletes.

##### 4.2.2.2 Multi-omics for injury risk assessment

The combination of AI with multi-omics biomarkers possesses transformative promise for the precise prevention of exercise-related ailments. OMICS technologies provide unparalleled insights into individual vulnerability, recovery, and performance enhancement, although their complete potential is achieved through AI-driven analytics. Machine learning algorithms may evaluate many data sources including metabolic pathways, gene expression patterns, protein biomarkers, genetic predispositions, and real-time physiological data from wearable sensors—to construct precise predictive models for injury risk assessment. This integration goes beyond biological knowledge to enable personalised intervention and real-time workout, health, and recovery monitoring (6). However, no prospective trial has yet demonstrated that a multi-omics AI model reduces injury incidence compared with standard monitoring. The current evidence consists entirely of retrospective association studies and small pilot projects.

Collagen-related gene markers (*COL1A1* rs1800012, *COL5A1* rs12722) are linked to tendon and ligament injuries, matrix metalloproteinase genes (*MMP3* rs679620, *MMP3* rs591058) to extracellular matrix remodelling, and inflammation-related genes (*IL6*, *ACE*) to muscle damage and recovery. For complex traits like ACL rupture susceptibility and muscle strain risk, polygenic risk scores (PRS) that include the cumulative effects of several low-impact variations are better predictors than single-gene markers (6).

##### 4.2.2.3 Saliva-based multi-omics for real-world monitoring

The current advancement in multi-omics integration is the emergence of non-invasive monitoring technologies. A laboratory crossover trial with 24 h sampling (413 saliva samples) indicated that saliva-based targeted multi-omics, which measures proteins, metabolites, and lipids, offers excellent analytical reliability and distinct molecular individuality. The algorithm utilized machine learning on single-timepoint saliva samples to successfully predict recent physical exercise both immediately and 24 h post-exercise. This analytical framework was applied in a longitudinal study of elite football players over 16 months, involving more than 12,000 saliva samples. Notwithstanding heightened biological and environmental variability, the model maintained strong differentiation between exercise and rest. The authors presented a saliva-based internal strain metric that quantifies internal load and can be utilized to track physical training and recovery, providing a scalable, non-invasive framework for physiological monitoring in athletes (137). While innovative, this approach requires validation in independent cohorts and demonstration that predictive models incorporating saliva omics outperform wearable-only models before it can be considered for clinical deployment.

##### 4.2.2.4 Sportomics paradigm

Muniz-Santos et al. (124) proposed the “sportomics” concept to understand multilevel metabolism while exercise in real life. Sportomics allows tailored research, including n-of-1 clinical trials and long-term athlete follow-up. Sportomics could use Big Data from top athletes to research human diseases, especially through nontargeted analysis. AI, Bioinformatics, and integrative computational analysis work together to study biochemical processes and identify biomarkers, including doping control biomarkers.

In spite of these developments, there are still numerous obstacles. Generalizability is restricted by the fact that the majority of multi-omics studies are based on relatively small cohorts, which are mostly made up of Caucasian or sport-specific populations. Before clinical translation, multi-ethnic validation is needed, and genetic connections are probabilistic. Moreover, ethical and legal issues with data privacy, authorisation, and possible genetic data misuse hamper the application of precision sports medicine (1).

#### 4.2.3 Critical appraisal of integrated AI-genomics models

We critically evaluated five landmark studies that explicitly combine genomic and AI methodologies (10–12, 136, 137). Common limitations across all five include: (1) absence of external validation in independent athlete cohorts; (2) small sample sizes (*n* = 24 to *n* = 142); (3) lack of pre-registered analysis plans, increasing risk of data dredging; (4) failure to report confidence intervals or calibration metrics alongside AUC values; and (5) no comparison against simple baseline models (e.g., logistic regression using only age, sex, and training history). These gaps illustrate that AI-genomics integration in sports remains at the “proof of concept stage”.

### 4.3 Systems biology approaches to understanding athletic performance

#### 4.3.1 Network-based modeling of biological pathways influencing performance

In sports research, systems biology represents a paradigm shift from reductionist analyses of individual molecular markers to thorough, network-based modelling of complex biological systems that underpin athletic performance (124). Athletic performance is influenced by molecular, multicellular, and extracellular matrix dynamics in the musculoskeletal, neuroendocrine, and cardiovascular systems. Network-based modelling captures this complexity and reveals crucial regulatory hubs and pathways that impact performance characteristics by representing biological molecules as nodes and their functional connections as edges.

The PhenoMol bioinformatic framework uses graph theory and biological knowledge to combine phenotypic data with multi-omic dimensionality reduction. Instead of using individual molecular markers, PhenoMol uses molecular interaction databases to create physiologically informed “expression circuits”, groups of molecules that cooperatively identify high-performing subgroups (136). Biomarkers were categorised into three cellular pathways: blood coagulation and complement cascade, which aid tissue injury recovery; urea cycle, which removes ammonia from protein catabolism; and mitochondrial function, which generates energy (136).

Using the HERITAGE Family Study dataset, Clarke et al. (138) used data-driven reverse engineering to identify gene regulatory networks directing skeletal muscle adaptation to endurance exercise. Endurance training modified the network to include 8,893 genes in 25 clusters. Fewer than 11% of these genes were differentially expressed by standard methods. Eif6 is the most linked hub, as seen in Eif6 haploinsufficient mice (Eif6⁺⁄⁻) with altered mitochondrial respiration, increased reactive oxygen species, and reduced exercise capacity.

Nevertheless, network-based models are highly sensitive to input data quality and prior knowledge biases. If molecular interaction databases are incomplete or tissue-specific (e.g., using generic protein-protein interaction networks rather than muscle-specific networks), inferred pathways may be biologically misleading.

#### 4.3.2 Holistic understanding of genotype–phenotype relationships

Systems biology in sports science aims to go beyond single-gene association studies to genotype–phenotype mapping. Athletic performance is a multifaceted phenotype that cannot be elucidated by singular genetic variants but arises from the interaction of numerous DNA polymorphisms, epigenetic alterations, environmental influences, and their interactions (17).

Sportomics is a top-down approach to analysing dynamic molecular and biochemical changes during sports activities and exercise. Sportomics amalgamates many omics disciplines, genomics, epigenomics, transcriptomics, proteomics, and metabolomics into a cohesive framework for the systematic examination of sports-induced alterations across all biological levels. In contrast to hypothesis-driven research, sportomics utilizes a non-hypothesis-driven approach, facilitating the discovery of novel biomarkers without assumptions (124).

Sports genomics has shown that DNA SNPs correlate with athletic performance and levels, influencing endurance, strength, power, speed, metabolic fitness, and psychological characteristics. The intricacy of sports phenotypes necessitates comprehensive models that include the emergent characteristics of biological systems (124).

Regulatory non-coding RNAs, including microRNAs (miRNAs), long non-coding RNAs (lncRNAs), and circular RNAs (circRNAs), are synthesized by approximately 99% of the human genome, which does not encode proteins. In exercise, miRNAs, such as miR-206, miR-133a, and miR-486, serve as dynamic interfaces that link genetic predisposition with environmental factors, such as nutrition and training intensity (1).

However, holistic understanding is currently constrained by the fact that approximately 99% of the human genome is non-coding, and the functional significance of most regulatory variants in exercise contexts is unknown. While microRNAs serve as dynamic interfaces linking genetic predisposition with environmental factors, causal evidence that manipulating these miRNAs improves performance or recovery is absent.

Although progress has been made, obstacles persist. The generalizability of the majority of multi-omics studies is restricted by their reliance on small, predominantly Caucasian cohorts. Approximately 80% of the 155 genetic markers that are purportedly linked to athletic performance have not been replicated. Large collaborative initiatives with standardized phenotyping and the integration of multiple omics layers are necessary for future research (1).

### 4.4 Incremental value of genomic data in AI models: a critical appraisal

A crucial question for practical implementation is whether genomic information meaningfully improves prediction or decision-making compared with established sports science variables. Existing models already incorporate training history, physiological characteristics, performance progression, injury history, anthropometry psychological status, and environmental factors. We identified only two studies that explicitly compared models with and without genetic features: (1) Wu et al. (9) found that adding 13 SNPs to a running-injury model improved AUC from 0.71 to 0.78, but the improvement was not statistically significant (*Δ*AUC 0.07, 95% CI −0.02 to 0.16); and (2) Varillas-Delgado et al. (139) found that + training load model showed no improvement over training load alone for muscle injury protection. We conclude that, at present, genomic data provides limited incremental predictive value beyond well-measured conventional variables. Its primary near term utility may lie in hypothesis generation and mechanistic insight rather than clinical prediction.

## 5 Personalized training and performance optimization in athletes

### 5.1 Genotype-based training strategies for enhancing athletic performance

#### 5.1.1 Personalized strength and endurance training adaptations

The idea of genotype-based training signifies a transition from uniform exercise regimens to personalized approaches that correspond with an athlete's genetic predisposition. A study by Jones et al. (140) formulated and validated a genetic algorithm for individualized resistance training, utilizing a panel of 15 performance-related gene polymorphisms. In two independent cohorts of male athletes (study 1: *n* = 28 athletes from different sports; study 2: *n* = 39 soccer players), participants undertook an eight-week resistance training regimen of either high or low intensity, which matched or did not match to their individual genotype. Athletes in the matched groups-those with power genotypes undergoing high-intensity training and those with endurance genotypes engaged in low-intensity training—exhibited substantial enhancements in Countermovement jump (CMJ), *P* = 0.0005) and aerobic 3 min cycle test performance (Aero3, *P* = 0.0004). In contrast, athletes in mismatched groups exhibited non-significant enhancements in CMJ (*P* = 0.175) and less notable outcomes in Aero3 (*P* = 0.0134). In both trials, 82% of non-responders or low responders belonged to mismatched groups (*P* < 0.0001), indicating that aligning individual genotype with the suitable training modality results in more successful resistance training outcomes. However, this study enrolled only 67 athletes total across two cohorts, was not prospectively validated, and has not been independently replicated. The effect sizes, while statistically significant, may reflect regression to the mean or selection bias rather than genuine genotype-training interactions. It should be considered a proof of concept requiring much larger replication, not a validated protocol.

Massidda et al. (141) developed a genetic model that includes six polymorphisms (*ACTN3*, *BDKRB2*, *ACE*, *VDR*-*ApaI*, *VDR*-*BsmI*, and *VDR-FokI*) to estimate vertical jump performance in 90 elite Italian soccer players. The Total Weighting Genotype Score (TWGS), an innovative algorithm that considers the proportion of significant phenotypic variance attributed to each polymorphism, indicated that only three polymorphisms (*ACTN3*, *ACE*, and *BDKRB2*) were included in the regression equation, accounting for 17.68–24.24% of the variance in vertical jump performance. This model facilitates the growth of personalized training programs specific to genetic predispositions and identifies athletes that necessitate modified training schedules to address injury vulnerabilities.

Bottura & Dentillo (142), reported on genomic-based decision-making in a 23-year-old competitive Brazilian open water marathon swimmer. Twenty genetic polymorphisms were examined to advise strength training periodization and endurance capability. Tailored interventions and longitudinal training load monitoring increased athletic performance and qualified athletes for the Absolute World Championships. Genetic-based training with organized load monitoring can optimize high-performance athlete preparation, as shown in this case. Single-case reports cannot establish generalisable efficacy and are susceptible to placebo effects and publication bias.

Comparing these three genotype-based training studies reveals a gradient of evidence quality. Jones et al. (140) (*n* = 67) is the most rigorous, with a controlled design and replication across two cohorts, yet remains underpowered and unvalidated. Massidda et al. (141) (*n* = 90) used a more sophisticated scoring algorithm but did not test training matching prospectively. Bottura & Dentillo (142), is purely anecdotal. None demonstrated that genotype-matched training produces superior long-term outcomes (e.g., reduced injury, sustained performance improvement) compared with standard periodisation. The modest explained variance (17%–24% for TWGS) indicates that genetic factors account for only a fraction of training response, with environment, psychology, and coaching quality likely dominating. Consequently, genotype-informed training prescription holds promise as a complementary approach to evidence-based coaching, with further research needed to establish its practical application.

#### 5.1.2 Genotype-specific exercise prescription

In a review of genetic and epigenetic determinants of training adaptation (1), SNPs in collagen-related genes (*COL1A1*, *COL5A1*), muscle structure genes (*ACTN3*), and inflammatory regulators (*IL6*) were found to modulate training Polygenic risk scores (PRS), which combine the effects of numerous low-impact variants, predict training adaptation and injury susceptibility better than single-gene indicators. However, PRS for training adaptation and injury susceptibility have not been externally validated and currently lack the predictive accuracy required for clinical use. Exercise-responsive microRNAs (e.g., miR-206, miR-133a, miR-486) provide adaptive training prescription theoretically, but causal evidence is absent.

### 5.2 Artificial intelligence–driven personalized training recommendations

#### 5.2.1 Real-time monitoring using wearable technologies

The combination of wearable technologies and artificial intelligence has enabled unparalleled real-time assessment of athlete physiology and performance. Bedrač et al. (143) reveals that continuous glucose monitoring (CGM) has become an essential tool for endurance athletes, with eleven studies utilizing CGM to evaluate the metabolic responses during training. Combining wearable sensors with machine learning algorithms allows real-time decision-making by detecting athlete-specific physiological reactions.

Modern wearables and mobile sensors can analyse biomarkers like sweat metabolites in real time, providing hydration and substrate consumption data. Combining digital platforms with biological data overcomes major limitations in conventional nutritional monitoring, allowing athletes and support teams to make rapid hydration and diet changes. Penggalih et al. (144) claim that wearable technologies and machine learning algorithms improve decision-making by recognising unique metabolic responses in each athlete, enabling the formation of personalised training and nutrition plans. However, current FDA/CE-approved wearable AI systems in sports are limited to load monitoring and do not yet incorporate genomic or multi-omics data streams.

#### 5.2.2 Adaptive and feedback-driven training algorithms

AI-powered systems are improving at giving adaptive, feedback-oriented training recommendations that improve with athlete success. The Maxiom platform uses an adaptable, human-centered intelligence engine based on peer-reviewed science and massive data sets to make personalised suggestions that expand with new research, biomarkers, and wearable data. The system delivers customised training and recuperation information, DNA-informed nutrition programmes, stress management, sleep enhancement, and injury prevention resources, and adaptive feedback from real-time data (145).

Accurate load control, quick recovery, and real-time monitoring are made possible by the integration of genetics, wearable technologies, and artificial intelligence. According to Ding et al. (1), several next-generation models that combine wearable biosensing, pharmacogenomic, genomic, and multi-omics profiling have been proposed for the best individualisation of athlete performance, recovery, and injury prevention strategies in top sports. Functional variability in metabolites such as 4-hydroxyproline, methionine, oxaloacetate, and tyrosine, as well as differential expression of proteins associated with immune response, muscle damage, metabolic fitness, and hemostasis, have been shown in multi-omics data from actual training conditions. These findings provide molecular signatures of adaptation and recovery that can direct adaptively training algorithms.

Comparing adaptive training platforms reveals a spectrum from research prototypes to commercial products. Zhang et al.'s (113) DQN (*n* = 25) and Zhou & Zhou's (114), Markov model (simulated data) represent academic proofs-of-concept with limited athlete testing. The Maxiom platform, while commercially available, lacks independent peer-reviewed validation. No study has prospectively compared AI-adaptive training against standard periodisation in a randomised controlled trial. The absence of head-to-head efficacy data means that claims of “personalised optimisation” remain marketing assertions rather than evidence-based conclusions.

### 5.3 Precision nutrition and recovery strategies in sports performance

#### 5.3.1 Nutrigenomics and individualized dietary planning

Precision sports nutrition now relies heavily on nutrigenomics, which studies how genetic variations and nutrients interact with athletic performance and recuperation. Sivasathiya et al. (146) identified key genetic variations that impact nutrient metabolism in athletics. The *CYP1A2* genotype influences endurance, recovery, oxygen transport, caffeine responses, iron metabolism, and vitamin D pathways. Polymorphisms in *VDR* and *SLC23A1* influence vitamin D and C bioavailability, which impacts immunological function, antioxidant defence, and bone health during severe training.

A study by Bedrač et al. (143) found 10 nutrigenetics research on endurance athletes, mostly utilising candidate gene approaches to analyse genetic differences and performance Genetics can account for up to 70% of athlete status variance, highlighting genomics' importance in athletic potential. Genome-wide association studies have discovered multiple performance-related SNPs, but additional study is needed to provide dietary recommendations.

However, nutrigenetic advice in sports currently rests on candidate gene studies with small effect sizes. Genome-wide association studies have discovered multiple performance-related SNPs, but the field lacks randomised controlled trials showing that genotype-based diets improve athletic outcomes compared with standard sports nutrition.

With over 50,000 SNPs influencing cellular processes, athlete-specific diets can consider genetic predispositions affecting recovery, metabolism, and training adaptations. Vitamin D, iron, and folate polymorphisms may necessitate customised diet for sportsmen to maintain optimal physiological parameters. Penggalih et al. (144) suggest that nutrigenomics can reveal genetic susceptibilities to nutritional deficiencies and enable customised supplementation to improve performance and recovery.

#### 5.3.2 Biomarker-guided recovery and fatigue management

Exercise-sensitive microRNAs may influence rehabilitation techniques. Ding et al. (1) state that miR-206, miR-133a, and miR-486 are essential epigenetic regulators that relate genetic predisposition to diet and training load. These miRNAs affect inflammation, muscle regeneration, and mechanical stress adaption, making them useful but unexplored markers for individualised recovery evaluation.

Oxaloacetate and tyrosine, which are needed for energy production and mitochondrial activity, have changed significantly during aerobic exercise, according to metabolomic profiling. Myoglobin and creatine kinase, essential for muscle repair and adaptation, are overexpressed during exercise, proteomic study shows. Myoglobin supplies muscle cells with oxygen, whereas creatine kinase repairs and retains energy. Changes in the urine metabolome during intense exercise are important markers of recovery phases; decreased levels of specific metabolites highlight the physiological stress brought on by high activity and provide markers for specific recovery strategies (144).

Combining multi-omics approaches, such as proteomics, metabolomics, and genomics, allows for comprehensive monitoring of athletes' physiological responses, which enhances the precision of recovery recommendations. This allows for customised athlete profile and intervention strategies. Functional variability in particular metabolites and differential protein expression linked to immune response, muscle injury, metabolic fitness, and hemostasis have been shown by multi-omics data collected under actual training conditions. These findings suggest biomarker-guided recovery methods based on adaptation and recovery molecular markers (1, 143).

While these molecular signatures are scientifically interesting, no prospective study has demonstrated that biomarker-guided recovery protocols reduce injury rates or improve performance more effectively than standard protocols (e.g., periodised rest, subjective wellness scores, and load management).

Protein and carbohydrate-rich post-exercise nutritional treatment reduces muscle protein breakdown and promotes glycogen resynthesis. Salivary proteome profiling, which shows increases in total salivary proteins after continuous exercise, is a non-invasive way to monitor physical stress and recovery. Combine these non-invasive monitoring methods with AI-driven analytics to analyse recovery level and fatigue management in real time without a blood sample (144).

Comparing nutrigenomics and biomarker-guided recovery studies reveals a consistent pattern: mechanistic plausibility exceeds clinical evidence. Nutrigenomic associations (e.g., *CYP1A2*-caffeine, *VDR*-vitamin D) are biologically coherent but have not been tested in RCTs of genotype matched diets. Biomarkers-guided recovery protocols (using CK, cortisol, or multi-omics) correlate with training load but have not been shown to reduce injury incidence or improve performance when used to guide recovery decisions. The gap between molecular correlation and clinical utility remains the central challenge for precision nutrition in sports.

## 6 Injury risk prediction and prevention in athletes

### 6.1 Genetic determinants of injury susceptibility in sports

#### 6.1.1 Role of collagen-related genes

Maintaining musculoskeletal tissue integrity requires collagen-encoding genes. A thorough review of 26 research involving over 7,000 athletes analysed genetic variants with common sports injuries including ACL rupture, Achilles tendinopathy, and stress fractures (47).

*COL1A1*, which encodes type I collagen, has been extensively studied. The South African cohort does not have ACL rupture cases with the TT genotype, showing reduced tissue sensitivity to tensile loading, which protects against ACL rupture (47). The *COL5A1* gene, which produces collagen fibrils, is one of the risk factors. Sun et al. (147) found that the TT genotype of *COL5A1* rs13946 was associated with ACL injury (OR = 1.29, 95% CI [1.06, 1.58]), notably in Caucasians. The *MMP3* gene, which encodes matrix metalloproteinase-3, impacts tissue remodelling, and the rs679620 variation has inconsistent injury risk associations with *COL5A1* polymorphisms (47).

An odd ratio of 1.29 indicates a very weak association with poor discriminative ability for individual prediction. Furthermore, the *COL5A*1 rs12722 variant explains <2% of ACL rupture variance, and polygenic scores for soft tissue injury have not been externally validated. Association does not equal prediction: case-control studies identify group-level differences, whereas clinical utility requires prospective identification of which athletes will suffer injury.

#### 6.1.2 Genetic factors associated with inflammation and tissue repair

The *ACTN3* R577X polymorphism, which encodes *α*-actinin-3 in fast-twitch muscle fibres, may lead to muscle damage. Research found that individuals with the XX genotype have a higher risk of muscular and soft tissue injuries, suggesting that *α*-actinin-3 deficiency affects muscle fibre integrity (47). Varillas-Delgado et al. (139) found a substantial association between the TT genotype and greater injury severity (OR = 2.55; 95% CI 1.23–5.30), but no consistent correlate with injury occurrence. This association has not been replicated in independent prospective cohorts and should not be used to justify training modifications for individual athletes.

### 6.2 Biomarkers associated with injury risk, stress, and fatigue

#### 6.2.1 Creatine kinase, cortisol, and inflammatory cytokines

The most extensively studied biomarker for muscle damage is creatine kinase, which shows a significant increase 24 to 72 h after exercise or competition. CK is essential for detecting muscle stress and guiding load control despite considerable individual heterogeneity. The testosterone-to-cortisol ratio is a better indicator of training strain than either hormone alone, according to research on cortisol and testosterone as hormonal indicators of metabolic stress. Inflammatory markers, such CRP and IL-6, show tissue damage and immunological activity; prolonged increases suggest cumulative physiological stress throughout congested fixture times (148).

#### 6.2.2 Physiological indicators of overtraining and recovery status

An essential immunological biomarker is now salivary immunoglobulin-A (s-IgA). The risk of upper respiratory tract infections increases when exercise intensity increases because s-IgA levels decrease. Within two weeks, a decrease of more than 65% significantly increases the risk of disease (148). Heart rate variability (HRV) is recognized as a significant early predictor of injury risk. L. Rossi & Rodrigues (58), discovered that machine learning models attained significant predictive performance (AUC up to 0.86) by utilizing parameters such as sleep disturbances, heart rate variability, and stress as preliminary indications of injury risk. However, this AUC was derived from internal validation on 31 triathletes; whether the model maintains accuracy in other sports, sexes, or competitive level is unknown.

### 6.3 Artificial intelligence–based models for injury prediction and prevention

#### 6.3.1 Monitoring training load and biomechanical stress

L. Rossi & Rodrigues (58), recommended a synthetic data generation framework for triathlon training, revealing that machine learning models (LASSO, Random Forest, XGBoost) get an AUC of up to 0.86 by utilizing HRV, sleep quality, and stress as pivotal early indicators of injury. By combining gait biomechanics with training load measurements, a hybrid machine learning approach for predicting Achilles tendinopathy in runners achieved a ROC AUC of 0.91 and an F1-score of 0.56, greatly outperforming single-modality models. The F1-score of 0.56 indicates poor precision-recall balance, meaning many false positive would occur if the model were used clinically. High AUC combined with low F1 suggests the model discriminates poorly at clinically useful probability threshold.

#### 6.3.2 Predictive analytics and early warning systems for injury prevention

In comparison to traditional physiotherapy evaluation (AUC 0.71), a multicenter observational study evaluating an AI-driven performance monitoring system in 160 competitive athletes found a significantly higher predictive accuracy for injury risk (AUC 0.89), with the AI model showing superior sensitivity (86%) and specificity (82%) (67). By identifying slight physiological changes that anticipate injuries, wearable devices increase the accuracy of injury prediction (58). While promising, this was a single-centre observational study without randomisation or blinding. The control (physiotherapy evaluation) is not a standard predictive model but a clinical assessment, making the comparison non-equivalent. Independent replication with pre-registration is required.

Comparing injury prediction models exposes a consistent disconnect between statistical performance and clinical utility. L. Rossi & Rodrigues (58), (AUC 0.86, *n* = 31, internal validation) and the multicenter study (AUC 0.89, *n* = 160, single-centre) both report promising discrimination but lack external validation and prospective outcome assessment. The Achilles tendinopathy model (AUC 0.91, F1 = 0.56) illustrates that high AUC can coexist with poor precision-recall balance, rendering the model clinically impractical due to excessive false positives. None of these studies randomised athletes to AI-guided vs. standard monitoring, meaning whether algorithmic prediction actually reduces injury incidence remains unproven. The incremental value of adding genomic or multi-omics features to wearable-based models has not been demonstrated.

## 7 Ethical, legal, and social implications of AI and genomics in sports

Sport genetics involves difficult ethical issues about human rights, privacy, equality, and autonomy. The key difficulties include confidentiality violations, data ownership, accidental results disclosure, and re-identifying anonymised genetic data (13). A detailed survey of 243 participants found that 4% of athletes refused DNA testing due to ethical concerns, compared to 19% of coaching staff. This shows that coaches and support personnel worry more about ethics than players (5). Many experts oppose genetic testing in adolescents and children due to ethical issues beyond professional competence, such as minors’ inability to give informed consent and the risk of lifelong psychological harm (48). CRISPR-Cas9 gene-editing presents ethical concerns, especially for germline changes. Mielżyńska et al. (149) suggest that these modifications may lead to heritable changes in future generations, creating “genetically enhanced athletes” with superior physical traits.

### 7.1 Genetic privacy and data ownership

Genomic data is inherently identifiable and cannot be fully anonymised. Unlike credit card numbers, genetic sequences are immutable and reveal ancestry, disease risk, and biological relationships. In sports, it remains unclear whether the athlete, club, or governing body owns genomic data (150). We recommend that athletes retain primary ownership of data, with clubs holding only time-limited, purpose-specific license.

### 7.2 Discrimination and athlete selection

Protecting athletes' privacy and confidentiality in collecting and storing genetic information is crucial to prevent discrimination or stigmatization (151).

### 7.3 Minors and informed consent

Predictive genetic testing for athletic performance in minors should not be offered outside research settings. Children cannot provide fully informed consent for tests with uncertain predictive value, and positive or negative results may cause lasting psychological harm or self-fulfilling prophecies (48). Parents and coaches should not have unrestricted access to a minor athlete's genetic profile.

### 7.4 Legal liability and algorithmic accountability

If an AI algorithm erroneously clears an athlete for return-to-play who subsequently suffers injury, liability may fall on the clinician, the software developer, or the institution-a “responsibility gap” that remains unresolved in sports law (152). We propose that sports organisations adopt “Genetic Data Stewardship Committees” analogous to institutional review boards, and that AI decision-support tools carry black-box warnings stating they are adjuncts to, not replacement for, clinical judgement.

Responsible sports genomics advancement requires strong governance and worldwide cooperation. For sports genetic research evaluation, the ACCE model (Analytical validity, Clinical validity, Clinical utility, and Ethical, legal, social implications) is recommended. Unlike clinical genetic testing for illness vulnerability, genetic testing for athletic ability is difficult to interpret and apply professional practice (13). The FIMS 2019 consensus declaration emphasises the need for scientists and clinicians to be ethical and data protection experts to develop the discipline without sacrificing athlete privacy (5). Finally, scientific development must respect athlete privacy, dignity, and individuality.

## 8 Limitations and challenges in AI and genomics applications in sports

### 8.1 Limited reproducibility of genotype–phenotype associations

One of the main challenges in sports genomics is the limited reproducibility of genotype-phenotype associations across various groups and research. Exercise genomics research has generally been hindered by small sample numbers and biassed techniques, resulting in erroneous correlations and exaggerated effects. As a result, existing genetic tests derived from these inherently constrained data cannot accurately predict athletic performance (44).

Statistical power continues to be a significant concern. To identify a meaningful link for a continuous variable with an SNP accounting 1% of variance among 500 tested SNPs, a minimum of 2,200 participants is required to achieve 80% power. For case-control studies with feasible odds ratios of 1.2 and a minor allele frequency of 20%, a sample size of 4,000 cases and 4,000 controls is necessary. Numerous sports genomics research significantly fail to meet these criteria (44).

### 8.2 Bias and heterogeneity in AI models and datasets

Applications of artificial intelligence in sports genomics encounter substantial obstacles associated with heterogeneity and bias. Konopka et al. (153) conducted a systematic SWOT analysis of exercise genetics, which revealed numerous deficiencies, including issues with causality, low generalizability, low research quality, complexity of exercise-related characteristics, and reporting bias. The overapplication of machine learning to inappropriate populations is a developing concern. Overuse of machine learning on unsuitable populations is a growing issue. According to G. Wang et al. (45), bias can show up as selection bias in athlete cohorts, confirmation bias in favourable outcomes, and population stratification (differences in allele frequencies attributable to ancestral background rather than genuine associations).

Bias can show up as selection bias in athlete cohorts, confirmation bias in favourable outcomes, and population stratification (differences in allele frequencies attributable to ancestral background rather than genuine associations) (45).

### 8.3 Small sample sizes and underrepresentation of populations

The most common limitation on the use of AI and genomics in sports is small sample numbers. Investigations into elite professional teams, particularly involving female athletes, are limited by participant accessibility, high costs, and the logistical difficulties associated with prolonged investigations in competitive sports. Even in research with nominal sample sizes as low as 24 participants, longitudinal methodologies employing repeated daily observations can produce effective sample sizes in the hundreds. However, such approaches cannot completely compensate for lower participant numbers when evaluating population-level genetic effects (154).

There are some methodological problems with this research, like not being able to measure true exercise performance phenotypes, which makes it hard to understand the study results. Also, many studies don't use enough athletes or aren't sure if the athletes they do include are top, which could lead to expectancy effects (18).

Underrepresentation of various groups makes it very hard to generalize. Almost all studies on sports genomics have been done on Caucasian populations, with few players of African, Asian, or Hispanic descent being included. Future research must examine the similarities and differences between racial and ethnic groups as well as between sexes to establish broadly valid and repeatable connections (44).

### 8.4 High costs, technical complexity, and data integration challenges

The amalgamation of multi-omics data with AI-driven analytics poses significant technological and economical challenges. Untargeted metabolomics encounters substantial problems, such as the reproducibility of results, complexities in identification of metabolites, and the translational divide between extensive datasets and implementable nutritional strategies (155). Typically, only a small proportion of identified features can be accurately recognized due to constraints in spectrum libraries and reference standards.

However, the curse of dimensionality continues to be a fundamental obstacle, as the number of measurable molecular features significantly exceeds the number of participants in even the most extensive studies. The identification of statistically significant predictors of high performance is significantly restricted by the combination of multiple testing corrections and typically modest effect sizes (136). The field's additional vulnerabilities were identified by the SWOT analysis, which included high costs and invasive methods (153).

In response to Lolli, it was highlighted that building prognostic models from small sample sizes might lead to projections that are incorrect because of a lack of data. Wider deployment is made more difficult by the requirement that each team calibrate prediction models using their own data for best (154).

### 8.5 Methodological pitfalls in sports AI: overfitting, data leakage, and validation failure

This section addresses critical methodological concerns raised regarding AI model reliability. First, overfitting is rampant in small athlete cohorts (*n* < 100) with high dimensional omics data (the *P*>>N problem), where models memorise noise rather than learning generalisable patterns. Second, data leakage occurs via inappropriate cross-validation-such as using future data to predict past injuries, including multiple observations from the same athlete in both training and test sets, or selecting features before cross-validation. Third, external validation is absent in most cited studies: only 3 of 15 studies in Table 3 used independent cohorts. Fourth, reproducibility crises arise from non-shared code and proprietary wearable algorithms. Finally, generalizability is severely limited: models trained on male Caucasian endurance athletes fail when applied to female African sprint athletes. High predictive performance within a specific dataset does not necessarily indicate practical usefulness.

### 9 Future perspectives on AI and genomics in sports science

The future of AI and genomics in sports science lies in the integration of wearable biosensors, multi-omics technologies, and advanced artificial intelligence to develop increasingly individualised, predictive, and preventive athlete management systems. Several technologies have already demonstrated translational potential, whereas others remain at an early stage of research and require further validation before routine implementation in sports practice.

### 9.1 Technologies approaching practical implementation

AI-assisted analysis of genomic data, wearable sensors for monitoring physiological parameters (e.g., heart rate variability, sleep quality, and training load), and polygenic risk score (PRS)-based models are among the most advanced developments. These approaches shown promising results for monitoring training responses, optimising recovery, estimating injury susceptibility, and supporting precision load management, although their predictive accuracy and generalisability still require validation across diverse athlete populations (1). Similarly, Khoramipour et al. (156) proposed the concepts of enduromics and resistomics, which extend conventional sportomics by characterising the molecular adaptations associated with endurance and resistance training. While these frameworks provide valuable insights into exercise-induced biological responses, their widespread clinical and sporting application will depend on standardised protocols and large-scale validation studies.

### 9.2 Emerging research directions

Several promising technologies remain largely experimental and have not yet reached routine application in elite sports. Single-cell multi-omics has the potential to reveal cell-specific molecular responses to exercise by simultaneously characterising gene expression, epigenetic regulation, protein abundance, and metabolic pathways, but its high cost, technical complexity, and limited longitudinal evidence currently restrict its use to research settings (156). Similarly, intelligent biosensors capable of continuously measuring sweat metabolites, interstitial glucose, or salivary multi-omics biomarkers offer the prospect of non-invasive, real-time physiological monitoring; however, challenges related to analytical accuracy, sensor standardisation, long term reliability, and clinical validation remain to be addressed. Advanced genomic deep-learning architectures incorporating uncertainty quantification and domain specific biological constraints may further improve the prediction of athletic performance and injury susceptibility, but these models require extensive external validation before they can be adapted for routine decision making (11). Similarly, athlete digital twins-dynamic computational models that simulate an athlete's physiological and molecular state-represent a promising future direction for personalised training optimisation and injury prevention. At present, however, digital twins remain primarily conceptual, with only early proof of concept studies demonstrating their feasibility.

For this future, multicenter partnerships with varied ethnic representation, standardised phenotyping techniques, robust ethical governance framework for genetic data security, and fair access in men's and women's sports are needed. Genomic data science, sports science, and ethics must collaborate, follow consent protocols, and respect athlete dignity and individuality to advance responsibly.

## 10 Search strategy

The literature was reviewed using PubMed, Scopus, and Web of Science, and SPORTDiscus to find relevant peer-reviewed articles on artificial intelligence, sports genomics, precision sports medicine, wearable technologies, and multi-omics methods. The search period covered 2014-202026. Boolean search strings were constructed for four thematic blocks: (“artificial intelligence” OR “machine learning” OR “deep learning”) AND (“sports” OR “athlete” OR “exercise”); (2) (“genomics” OR “sports genomics” OR “genetic marker” OR “SNP”) AND (“athletic performance” OR “exercise” OR “sports”); (3) (“precision medicine” OR “personalised training” OR “precision sports”) AND (“athlete” OR “sports science”); and (4) (“multi-omics” OR “transcriptomics” OR “metabolomics” OR “proteomics”) AND (“exercise” OR “athlete”).

Inclusion criteria: peer-reviewed English language original research articles, systemic reviews, and meta-analyses involving human athletes. Exclusion criteria: non-human studies, non-peer reviewed sources, and articles focusing exclusively on clinical disease genetics without athletic performance relevance. Full-text review was performed on potentially eligible articles. Studies were selected based on scientific significance, methodological integrity, innovation, and contribution to AI-driven personalised athlete management.

## 11 Conclusions

AI and genetics are revolutionising sports science with personalised training, injury prediction, and data-driven athlete management. However, this revolution remains largely aspirational. The combination of genetic profiling, wearable technologies, multi-omics data, and AI-driven prediction models might improve athlete performance, recovery, and health, but no AI-genomics tool is currently ready for routine clinical or coaching deployment without prospective, multi-site, externally validated trials. Recent study shows that genetic, physiological, metabolic, and environmental factors affect athletic performance, which can now be more comprehensively analyzed by modern computational approaches.

AI-powered solutions have significant potential in the fields of athlete classification, training modification, fatigue assessment and early injury diagnosis. However, low repeatability, small and ethnically homogenous datasets, ethical considerations, difficulty in absorbing large biological data, and the absence of demonstrated incremental predictive value of genomic data beyond conventional sports science variables still hamper more widespread acceptance. Progress in the future will depend on large collaborative research, standardised methods, more ethical governance, diverse athlete population, and rigorous adherence to reporting standards such as CONSORT-AI, SPIRIT-AI, and TRIPOD-AI. The integration of AI and genomes represents a significant advancement in precision sports medicine and the future of personalised athletic performance management, provided that scientific development respects athlete privacy, dignity, and individuality, and that claims about transformative potential are moderated by transparent acknowledgement of current limitations.
